# Science and technology breakthroughs to advance artificial cultivation of true morels

**DOI:** 10.3389/fmicb.2023.1259144

**Published:** 2023-08-21

**Authors:** Qi Zheng Liu, Cai Hong Dong

**Affiliations:** State Key Laboratory of Mycology, Institute of Microbiology, Chinese Academy of Sciences, Beijing, China

**Keywords:** *Morchella*, cultivation, breakthroughs, research direction, development tendency

Morels are highly prized and delicious edible mushrooms. Commercial cultivation has achieved success, and the outdoor cultivation area has quickly expanded to over 10,000 ha after 2018 in China, but unstable yields and reductions in productivity still occur often. The industry's development has been plagued by problems such as the identification of true Morels, a lack of understanding of the life cycle, disease control, nutrient requirements, the initial cost of production, and a lack of commercial varieties. A lack of scientific research in the field has hindered the further development of the morel industry. The future research on morels in the next decade is proposed, including systematic cognition, the microbiome, new equipment, and so on.

## Systematic understanding of *Morchella* and the ecological environment

Many factors affected the growth of *Morchella*, including nutrient substances, soil, plants, etc. (Liu et al., 2018; Papadaki et al., 2019; Evangelista et al., 2021; Tan et al., 2021a; Xu et al., 2022a). Therefore, for the upgrading of morel cultivation technology, we should consider it from the perspective of eco-environment integration, and thinking from a single factor should be avoided. A comprehensive evaluation of the impacts of water, air, and materials on the entire process of morel cultivation is necessary. Other than the seven elata clade species that are currently cultivated (Du, 2019; Wu et al., 2019), it is important to systematically screen all *Morchella* species to exploit the stress resistant resources in *Morchella*. Keeping on collecting wild *Morchella* resources and expanding the germplasm resource bank through the discovery of new species and strains is important. Many other factors should be taken into account, such as morphology and biogeography, DNA barcoding, molecular systematics, global diversity, etc. In summary, to achieve breakthroughs in morel cultivation technology, we should first establish a systematic understanding of *Morchella* and the ecological environment.

## Microbiome analysis will be contributed to understanding the cultivation process of *Morchella*

It has been reported that soil microbiome has an important influence on morel cultivation and the α-diversity level and community evenness among soil fungal taxa could affect the production of morels (Tan et al., 2021b; Yu et al., 2022; Zhang et al., 2023). However, the changes in microbial communities during field cultivation and their effects are still not well understood. The microbial community is very sensitive to the interference of abiotic environmental factors, including soil tillage, temperature, heavy metals, and so on (Hu et al., 2022; Nelson et al., 2022). Currently, the changes in the microbiome in morel cultivation soil after the intervention of abiotic factors are little known.

With the deepening of microbiome research (Benucci et al., 2019; Longley et al., 2019), a database of microorganisms related to morel cultivation should be established to better understand the interaction between *Morchella* and the environmental microbiome. At the same time, the influence of the microbial community on the life history of *Morchella* can also be explored, and the spawn production process of *Morchella* can be deeply analyzed (Yu et al., 2023). By researching the functions of various microorganisms in the soil microbial community, some beneficial microorganisms can be found with the potential to create a more productive and sustainable morel cultivation system, which can promote the growth of *Morchella*, improve the use efficiency of exogenous nutrients, and enhance the resistance of *Morchella* to environmental stress and pathogenic microorganisms.

## The establishment of a platform for data collection and communication will be helpful for improving the management of morel cultivation

At present, sensor technology has been widely used in the field of agriculture (Singh et al., 2020; Arrubla-Hoyos et al., 2022). The development of big data, artificial intelligence, and other technologies provides more rapid collection, analysis, storage, sharing, and integration of data (Jung et al., 2021; Xu et al., 2022b). The large-scale cultivation of *Morchella* has been ongoing for more than 10 years in China and many laboratories and companies have collected a large amount of data related to morel cultivation (Guo et al., 2020; Tan et al., 2021c). However, there is a lack of effective tools to analyze and use the existing data. The progress of data science and information technology can greatly improve the ability to solve complex problems and provide an important opportunity for improving the management technology of morel cultivation. Therefore, the establishment of a network platform that can store and open access morel cultivation data sets will be helpful for sharing data. Making full use of the latest information technology to accelerate the unification and standardization of morel cultivation technology is the key to improving the management of morel cultivation.

## Focus on basic research and embrace new breeding techniques

In recent years, basic research on *Morchella* has made some progress in terms of life cycle, sclerotium development mechanisms, exogenous nutrition utilization, characteristic of mitochondrial genome, etc. (Tan et al., 2019; Liu et al., 2020, 2021a; Du and Yang, 2021; Chai et al., 2022). However, there is still a lack of understanding of conidial production, working mechanisms of exogenous nutrition bags, primordium formation, and fruiting body development mechanisms (Liu et al., 2022), so basic research should be continued.

With the development of gene editing technology (Wang et al., 2019), improvement of *Morchella* strains by precise breeding is expected to be realized in the next few years. Advances in basic research combining with precise breeding methods can help to accurately and rapidly improve the biological traits that are important for the yield and quality of *Morchella*. This technology opens the door to the rapid development of superior *Morchella* strains.

## New equipments and materials will be the new driving force for the development of the *Morchella* industry

How to improve the labor efficiency of morel cultivation is also a problem that needs to be addressed. In recent years, automatic external nutrient bag packing machines, automatic strain bag breaking machines, and other small equipments have been applied in morel cultivation. On the other hand, the development of automatic morel fruiting body picking equipment and packaging equipment is of great significance for reducing labor consumption and transportation loss. New equipment-related research and development should be paid more attention.

Exogenous nutrition bags consume a lot of labor and may bring environmental pollution (Liu et al., 2017). Recent experiments have found that filter paper coated with exogenous nutrients can play the same role as polypropylene bags (Liu et al., 2021b), suggesting that it is possible for environmentally friendly materials to replace polypropylene plastics. Based on the basic principle of exogenous nutrient bags delivering nutrients to soil (Tan et al., 2019), more materials should be tried to replace plastics or make more efficient use of exogenous nutrients and reduce environmental pressure.

## Conclusions and future outlook

**Figure 1:**
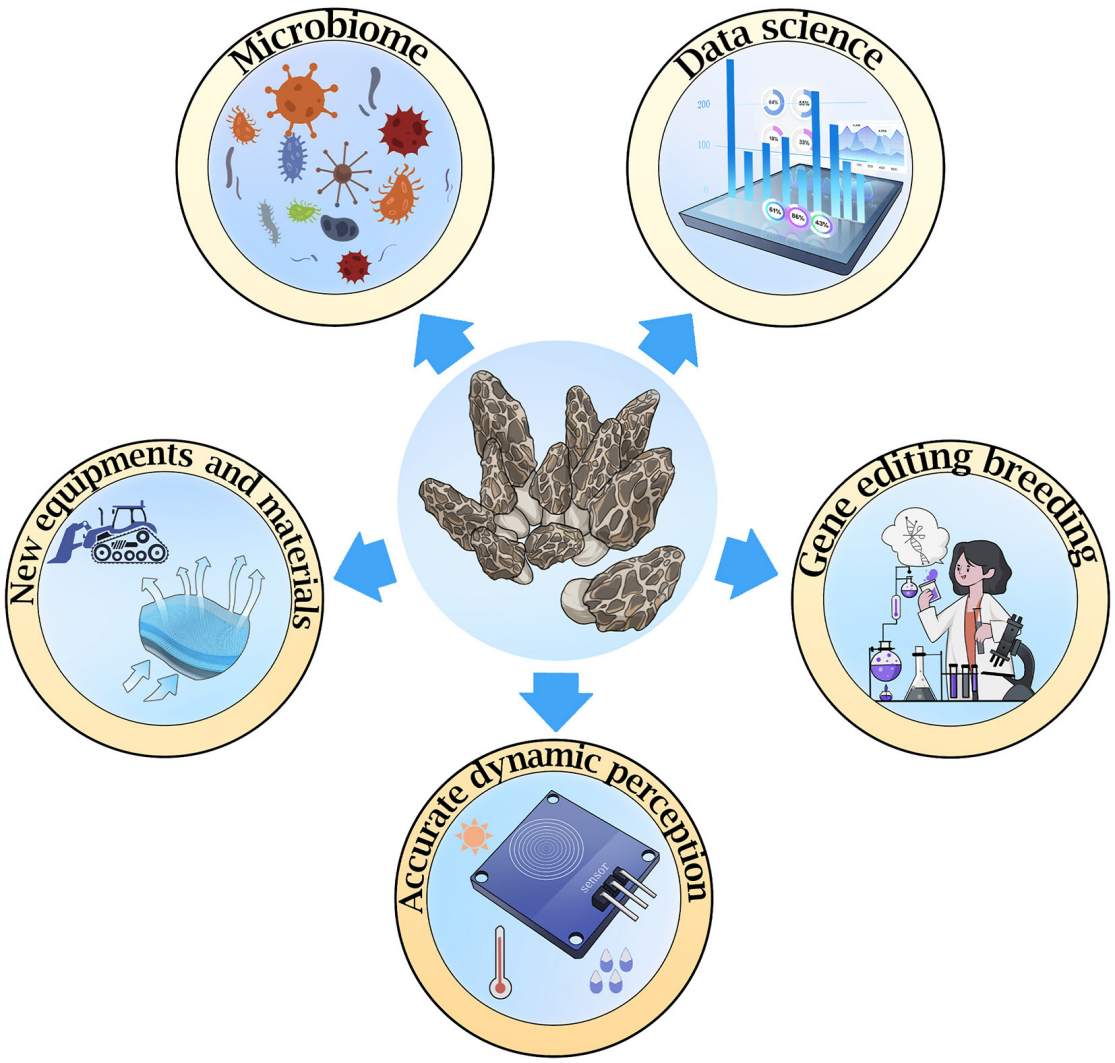
Five directions worth efforts in the development of *Morchella* cultivation.

In the next 10 years, we will seek scientific and technological breakthroughs in morel cultivation in five directions, including the microbiome, data science, accurate dynamic perception, new equipments and materials, and gene editing breeding (Figure 1). These are also key technologies that are indispensable in the morels field. It is expected that the scale of morel cultivation will continue to expand, not only in field cultivation but also in the scale of industrial cultivation. Advances in science and technology will lay the foundation for the development of the morels industry.

## Author contributions

QL: Conceptualization, Funding acquisition, Writing—original draft. CD: Supervision, Writing—review and editing.

## References

[B1] Arrubla-Hoyos W. Ojeda-Beltrán A. Solano-Barliza A. Rambauth-Ibarra G. Barrios-Ulloa A. Cama-Pinto D.. (2022). Precision agriculture and sensor systems applications in Colombia through 5G networks. Sensors (Basel) 22, 7295. 10.3390/s22197295 36236394 PMC9571140

[B2] Benucci G. M. N. Longley R. Zhang P. Zhao Q. Bonito G. Yu F. Q. (2019). Microbial communities associated with the black morel *Morchella sextelata* cultivated in greenhouses. PeerJ. 7, e7744. 10.7717/peerj.7744 31579614 PMC6766373

[B3] Chai H. M. Ma Y. H. Liu P. Chen W. M. Tao N. Zhao Y. C. (2022). The asymmetrical distribution of opposite mating type nuclei in single-ascospore isolates revealed *Morchella importuna* is a pseudohomothallic fungus. Mycosystema 1, 12. 10.13346/j.mycosystema.220035

[B4] Du X. H. (2019). Review on species resources, reproductive modes and genetic diversity of black morels. J. Fungal Res. 17, 240–251. 10.13341/j.jfr.2019.8016

[B5] Du X. H. Yang Z. L. (2021). Mating systems in true morels (*Morchella*). Microbiol. Mol. Biol. Rev. 85, e00220–e00220. 10.1128/MMBR.00220-20 34319143 PMC8483713

[B6] Evangelista F. R. Chairez I. Sierra S. Leal Lara H. Martínez-González C. R. Garín Aguilar M. E.. (2021). A novel coconut-malt extract medium increases growth rate of morels in pure culture. AMB Express 11, 167. 10.1186/s13568-021-01325-2 34910284 PMC8674397

[B7] Guo Y. X. Jiang Y. Q. Chen Q. J. Zhang G. Q. Chang J. F. (2020). Research on cultivation adaptability of *Morchella* in Beijing. J. Beijing Univ. Agric. 35, 33–39. 10.13473/j.cnki.issn.1002-3186.2020.0307

[B8] Hu D. W. Li S. S. Liu G. H. (2022). Research progress on the influence of the disturbance of abiotic environmental factors on the succession of microbial community. J. Beijing Univ. Aeron. Astron. 2022, 1–11. 10.13700/j.bh.1001-5965.2022.0736

[B9] Jung J. Maeda M. Chang A. Bhandari M. Ashapure A. Landivar-Bowles J. (2021). The potential of remote sensing and artificial intelligence as tools to improve the resilience of agriculture production systems. Curr. Opin. Biotechnol. 70, 15–22. 10.1016/j.copbio.2020.09.003 33038780

[B10] Liu Q. Z. He G. Q. Wei J. K. Dong C. H. (2021a). Comparative transcriptome analysis of cells from different areas reveals ROS responsive mechanism at sclerotial initiation stage in *Morchella importuna*. Sci. Rep. 11, 9418. 10.1038/s41598-021-87784-w 33941791 PMC8093252

[B11] Liu Q. Z. Ma H. S. Zhang Y. Dong C. H. (2018). Artificial cultivation of true morels: current state, issues and perspectives. Crit. Rev. Biotechnol. 38, 259–271. 10.1080/07388551.2017.1333082 28585444

[B12] Liu Q. Z. Qu S. He G. Q. Wei J. K. Dong C. H. (2022). Mating-type genes play an important role in fruiting body development in *Morchella sextelata*. J. Fungi. 8, 564. 10.3390/jof8060564 35736047 PMC9225556

[B13] Liu Q. Z. Qu S. Tan F. H. Dong C. H. (2021b). Influencing factors of exogenous nutrient in *Morchella importuna*. Mycosystema 40, 3157–3168. 10.13346/j.mycosystema.210329

[B14] Liu W. Cai Y. L. Zhang Q. Q. Chen L. F. Shu F. Ma X. L.. (2020). The mitochondrial genome of *Morchella importuna* (272.2 kb) is the largest among fungi and contains numerous introns, mitochondrial non-conserved open reading frames and repetitive sequences. Int. J. Biol. Macromol. 143, 373–381. 10.1016/j.ijbiomac.2019.12.056 31830457

[B15] Liu W. Cai Y. L. Zhang Y. Zhao X. Bian Y. B. (2017). Analysis of input-output in morels cultivation project in field. Edible Med. Mushr. 25, 220–225.

[B16] Longley R. Benucci G. M. N. Mills G. Bonito G. (2019). Fungal and bacterial community dynamics in substrates during the cultivation of morels (*Morchella rufobrunnea*) indoors. FEMS Microbiol. Lett. 366, fnz215. 10.1093/femsle/fnz215 31603508 PMC6836762

[B17] Nelson A. R. Narrowe A. B. Rhoades C. C. Fegel T. S. Daly R. A. Roth H. K.. (2022). Wildfire-dependent changes in soil microbiome diversity and function. Nat. Microbiol. 7,1419–1430. 10.1038/s41564-022-01203-y 36008619 PMC9418001

[B18] Papadaki A. Diamantopoulou P. Papanikolaou S. Philippoussis A. (2019). Evaluation of biomass and chitin production of *morchella* mushrooms grown on starch-based substrates. Foods 8, 239. 10.3390/foods8070239 31266266 PMC6678217

[B19] Singh R. K. Aernouts M. De Meyer M. Weyn M. Berkvens R. (2020). Leveraging LoRaWAN technology for precision agriculture in greenhouses. Sensors (Basel) 20, 1827. 10.3390/s20071827 32218353 PMC7181210

[B20] Tan H. Kohler A. Miao R. Y. Liu T. H. Zhang Q. Zhang B.. (2019). Multi-omic analyses of exogenous nutrient bag decomposition by the black morel *Morchella importuna* reveal sustained carbon acquisition and transferring. Environ. Microbiol. 21, 3909–3926. 10.1111/1462-2920.14741 31314937

[B21] Tan H. Liu T. H. Yu Y. Tang J. Jiang L. Martin F. M.. (2021b). Morel production related to soil microbial diversity and evenness. Microbiol. Spectrum. 9, e00229–e00221. 10.1128/Spectrum.00229-21 34643439 PMC8515941

[B22] Tan H. Miao R. Y. Liu T. H. Tang J. Du D. B. Han H. T.. (2021c). Ranges and dynamics of environmental factors for *Morchella importuna* cultivation in chengdu plain area. Southwest China J. Agric. Sci. 34, 27–39. 10.16213/j.cnki.scjas.2021.1.004

[B23] Tan H. Yu Y. Tang J. Liu T. H. Miao R. Y. Huang Z. Q.. (2021a). Build your own mushroom soil: microbiota succession and nutritional accumulation in semi-synthetic substratum drive the fructification of a soil-saprotrophic morel. Front. Microbiol. 12, 656656. 10.3389/fmicb.2021.656656 34108948 PMC8180906

[B24] Wang X. P. Zhang J. J. Dong C. H. (2019). Research progress of CRISPR/Cas9 mediated genome editing in edible and medicinal fungi. J. Fungal Res. 17,215–223. 10.13341/j.jfr.2019.801337303782

[B25] Wu F. Zhou L. W. Yang Z. L. Bau T. Li T. H. Dai Y. C. (2019). Resource diversity of Chinese macrofungi: edible, medicinal and poisonous species. Fungal Divers. 98, 1–76. 10.1007/s13225-019-00432-7

[B26] Xu Y. Tang J. Wang Y. He X. Tan H. Yu Y.. (2022a). Large-scale commercial cultivation of morels: current state and perspectives. Appl. Microbiol. Biotechnol. 106, 4401–4412. 10.1007/s00253-022-12012-y 35731306

[B27] Xu Y. Zhang X. Li H. Zheng H. Zhang J. Olsen M. S.. (2022b). Smart breeding driven by big data, artificial intelligence, and integrated genomic-enviromic prediction. Mol. Plant. 15, 1664–1695. 10.1016/j.molp.2022.09.001 36081348

[B28] Yu F. M. Jayawardena R. S. Thongklang N. Lv M. L. Zhu X. T. Zhao Q. (2022). Morel production associated with soil nitrogen-fixing and nitrifying microorganisms. J. Fungi. 8, 299. 10.3390/jof8030299 35330300 PMC8950353

[B29] Yu Y. Liu T. H. Liu L. X. Tang J. Peng W. H. Chen Y.. (2023). Study on aerosol microbial community in the production workshop of morel spawn. Biotechnol. Bull. 39, 267–275. 10.13560/j.cnki.biotech.bull.1985.2022-0702

[B30] Zhang Y. Sun S. Luo D. Mao P. Rosazlina R. Martin F.. (2023). Decline in morel production upon continuous cropping is related to changes in soil mycobiome. J. Fungi. 9, 492. 10.3390/jof9040492 37108946 PMC10143708

